# Weight loss from 20 years of age is associated with cognitive impairment in middle-aged and elderly individuals

**DOI:** 10.1371/journal.pone.0185960

**Published:** 2017-10-05

**Authors:** Kaori Kitamura, Yumi Watanabe, Kazutoshi Nakamura, Akemi Takahashi, Ribeka Takachi, Rieko Oshiki, Ryosaku Kobayashi, Toshiko Saito, Shoichiro Tsugane, Ayako Sasaki

**Affiliations:** 1 Division of Preventive Medicine, Niigata University Graduate School of Medical and Dental Sciences, Niigata, Japan; 2 Department of Physical Therapy, Niigata University of Rehabilitation, Murakami, Niigata, Japan; 3 Department of Food Science and Nutrition, Nara Women’s University Graduate School of Humanities and Sciences, Nara, Japan; 4 Department of Physical Therapy, Niigata University of Health and Welfare, Niigata, Japan; 5 Department of Health and Nutrition, Niigata University of Health and Welfare, Niigata, Japan; 6 Center for Public Health Sciences, National Cancer Center, Tokyo, Japan; 7 Murakami Public Health Center, Murakami, Niigata, Japan; Texas Technical University Health Sciences Center, UNITED STATES

## Abstract

**Background:**

Few empirical studies have been conducted to identify modifiable factors that may affect cognitive impairment in Japanese individuals. The present study aimed to clarify whether body mass and lifestyle are associated with cognitive impairment in Japanese middle-aged and elderly individuals.

**Methods:**

Subjects were 1814 community-dwelling individuals aged 44–79 years, all of whom were participants of the Murakami Cohort Study baseline survey conducted in 2011–2013. Cognitive function was assessed using the Mini-Mental State Examination (MMSE) in 2014–2016, and cognitive impairment, the outcome measure, was defined as an MMSE score <24. Predictor variables were body mass index (BMI), long-term weight changes from 20 years of age, and lifestyle factors, such as smoking, drinking, and physical activity levels, which were obtained from a self-administered questionnaire in the baseline survey. Covariates were sex, age, education level, and histories of stroke and diabetes. Multiple logistic regression analysis was used to calculate the adjusted odds ratios (ORs).

**Results:**

The prevalence of overall cognitive impairment was 6.2%. The adjusted ORs of cognitive impairment in the lowest (<[-4]kg) (OR = 2.70, 95%CI, 1.18–6.20) and second ([-4]-[0]kg) (OR = 2.37, 95%CI, 1.04–5.37) quintiles for long-term weight change were significantly higher than the reference 4th quintile ([+4]-[+7]kg). The adjusted OR in the highest quintile (≥[+8]kg) was 2.24 (95%CI, 0.99–5.04). Current BMI was not associated with cognitive impairment.

**Conclusions:**

Long-term weight loss is associated with cognitive impairment in Japanese middle-aged and elderly individuals. Because the present study was retrospective in nature, prospective studies should also be conducted for further characterization of this association.

## Introduction

With the acceleration in aging, the number of individuals with dementia is increasing as well. In Japan, the number of elderly people with dementia is projected to be 4,100,000 (11.3% of those aged ≥65 years) in 2020, and 4,700,000 (12.8%) in 2025 [1]; a 1.5% increase during the next five years is expected.

Compared to cancer, cardiovascular disease, and cerebrovascular disease, far less is known about modifiable risk factors for dementia, and this gap in our understanding must be bridged. A recent meta-analysis showed that low educational attainment and decreased physical activity are likely risk factors for dementia [2]. In Japan, some epidemiologic studies reported that dietary patterns [3,4] and green tea consumption [5,6] may protect against cognitive impairment. However, the exact influence of lifestyle factors other than physical activity remains somewhat unclear among Japanese individuals [7]. Consequently, more epidemiologic studies are needed to gain a comprehensive understanding of lifestyle-related factors associated with dementia or cognitive impairment.

One factor that may be correlated with dementia is body mass. In European and North American countries, a large body of evidence suggests that being overweight or obese can increase one’s risk toward developing dementia [8–10]. In contrast, some studies reported being underweight as a risk factor [8]; a hospital-based epidemiologic study conducted in Japan found that being underweight may be a risk factor for cognitive impairment [11]. The association between body mass and dementia or cognitive impairment remains unclear, however, and further elucidation in population-based studies is warranted.

The present study aimed to determine whether body mass and lifestyle are associated with cognitive impairment in population-based samples of middle-aged and elderly Japanese. We used the Mini-Mental State Examination (MMSE) to assess cognitive impairment as the outcome measure, because MMSE-assessed cognitive impairment is widely used as a proxy outcome of dementia in many epidemiologic studies.

## Materials and methods

### Participants

The present study subjects were participants in the Murakami Cohort Study, a population-based cohort study performed to determine risk factors for age-related diseases [12]. The baseline study of the Murakami Cohort Study was conducted in 2011–2013 [12], at which point physical and lifestyle data were obtained. We further conducted an additional investigation to evaluate cognitive function for a subgroup of the cohort in 2014–2016, when we recruited subjects from among those who participated in the Murakami Cohort Study, primarily at the annual health-check examination setting provided by the local governments of Murakami City, Sekikawa Village, and Awashimaura Village. A total of 1814 middle-aged and elderly individuals agreed to participate in the present study and underwent an examination of cognitive function in the same setting. Written informed consent was obtained from all subjects. The protocol of this study was approved by the Ethics Committee of Niigata University School of Medicine.

### Physical and lifestyle assessment

Lifestyle information was obtained from all subjects. A self-administered questionnaire, which was completed as part of the baseline study of Murakami Cohort Study, extracted information on demographic characteristics, histories of stroke and diabetes, current height and weight, and lifestyle. It also extracted information on body weight at 20 years of age and at one year before, and long-term weight change was calculated as the current weight minus the weight at 20 years of age. Demographic characteristics included age, sex, and education levels. Education levels were classified on a scale of 1–4, for which 1: junior high school graduate (junior high school level), 2: high school graduate (high school level), 3: graduate of a junior college or a vocational college (junior college level), and 4: university graduate or higher (university level). Body mass index (BMI) was calculated by dividing weight (kg) by height squared (m^2^). Physical activity levels were estimated by calculating metabolic equivalents (METs) hours/day, which were obtained by multiplying the time score spent on physical activities (/day) by its MET intensity [13]; namely, heavy physical work or strenuous exercise (4.5), walking or standing (2.0), sedentary (1.5), and sleep or others (0.9). The validity of the total METs score has been published previously [14]. For smoking habit, subjects were classified as a non-smoker, past smoker, or current smoker. For alcohol drinking, subjects were classified as a non-drinker (including past drinkers), rare drinker, or current drinker. Alcohol consumption was assessed for current drinkers; information on average frequency, amount, and types of drinks was obtained, and weekly ethanol consumption was calculated [15]. Alcohol consumption was classified into five categories: 1) Non-drinker or rare drinker (these two groups were combined, because there was no significant difference in ORs between them), 2) 1–149, 3) 150–299, 4) 300–449, and 5) ≥450 g ethanol per week. Consumption of green tea and coffee was classified as 1) <1 times/wk, 2) 1–6 times/wk, 3) 1–3 cups/day, and 4) ≥4 cups/day. Number of sleeping hours was also obtained. The lifestyle questionnaire used in this study was the same as that used in the Japan Public Health Center-based Prospective Study [16].

### Assessment of cognitive function

Cognitive function was assessed using MMSE [17], a brief, validated instrument commonly used to screen for dementia. MMSE scores range from 0 to 30, with lower scores indicating greater cognitive impairment. Cognitive impairment was defined as an MMSE score of <24 [18, 19]. Using this cutoff value, the area under the receiver operating characteristic curve of MMSE for dementia diagnosed based on the Diagnostic and Statistical Manual of Mental Disorders Third Edition Revised (DSM-III-R), a classification system for mental illnesses developed by the American Psychiatric Association, in a Japanese population has been reported to be as high as 0.980 [18].

### Statistical analysis

Normality was assessed for continuous variables. To analyze sex-dependent differences, the Student’s t-test was used for continuous variables, such as age, BMI, and METs score; the Wilcoxon test was used for MMSE score; and the chi-square test was used for education level. Simple and multiple logistic regression analyses were performed to calculate odds ratios (ORs) of predictor variables for cognitive impairment. METs scores were divided into quartiles for OR comparison. BMI and long-term weight changes were divided into quintiles. We first checked the association between the demographic variables of sex, age, education level, and histories of stroke and diabetes [20] (basic determinants of MMSE-assessed cognitive impairment) and prevalence of cognitive impairment, and then ORs calculated according to potential predictors were adjusted for covariates, including sex, age, education level, and histories of stroke and diabetes. P for trend was calculated by logistic regression analysis. SAS statistical package (Release 9.4, Cary, NC, USA) was used for all statistical analyses. P<0.05 was considered statistically significant.

## Results

Mean subject age was 68.5 years (SD, 6.6), and the overall prevalence of cognitive impairment was 6.2%. Mean BMI was 23.0 kg/m^2^, and percentages of overweight (BMI ≥25) and obese (BMI ≥30) individuals were 21.1% and 1.6%, respectively. Subject characteristics by sex are shown in Table 1; all variables showed significant sex-dependent differences.

**Table 1:** Subject characteristics (means or numbers) by sex.

Characteristics	Means (SD) or numbers (%)		P value
	Men	Women	
Age (years)	69.4 (6.2)	67.9 (6.7)	<0.0001
BMI (kg/m^2^)	23.2 (2.7, 727)	22.8 (3.1, 1,074)	0.0123
METs score (/d)	32.6 (13.7, 732)	29.5 (12.8, 1,080)	<0.0001
Education level			<0.0001
Junior high school	262 (35.7)	452 (41.8)	
High school	390 (53.2)	491 (45.4)	
Junior college	37 (5.1)	111 (10.3)	
University or higher	44 (6.0)	27 (2.5)	
MMSE score	27.2 (2.5)	27.7 (2.3)	<0.0001
(max, 30 points)	28.0 (median)	28.0 (median)	

SD, standard deviation; BMI, body mass index; MET, metabolic equivalent.

The ORs of cognitive impairment according to demographic factors are shown in Table 2. Sex, age, and education levels were significantly associated with the prevalence of cognitive impairment. Regarding education level, the junior high school level had a much higher OR than other education levels, and therefore we created a new dummy variable for education level, whereby 0 was junior high school level and 1 was all other levels. These dummy variables were used as covariates in the multiple logistic regression analysis.

**Table 2:** Odds ratios (ORs) of cognitive impairment (MMSE score <24) according to levels of demographic factors.

Demographic factors	Prevalence of cognitive impairment (%)	Unadjusted OR (95%CI)
Sex		P = 0.0037
Men	60/733 (8.2)	1.76 (1.20–2.59)
Women	52/1081 (4.8)	1 (reference)
Age (years)		P<0.0001
<59	3/167 (1.8)	1 (reference)
60–69	31/809 (3.8)	2.18 (0.66–7.21)
70–79	78/838 (9.3)	5.61 (1.75–17.99)
Education level		P<0.0001
Junior high school	81/714 (11.3)	1 (reference)
High school	26/881 (3.0)	0.27 (0.17–0.44)
Junior college	4/148 (2.7)	0.35 (0.12–1.00)
University or higher	1/71 (1.4)	0.12 (0.02–0.89)
Self-reported history of stroke		P = 0.6564
No	109/1776 (6.1)	1 (ref)
Yes	3/38 (7.9)	1.31 (0.40–4.33)
Self-reported history of diabetes		P = 0.5038
No	102/1681 (6.1)	1 (ref)
Yes	10/133 (7.5)	1.26 (0.64–2.47)

CI, confidence interval.

ORs of cognitive impairment according to predictor variables are shown in Table 3. BMI was not associated with cognitive impairment. Regarding long-term weight changes, the adjusted ORs of the lowest and second quintiles were significantly higher than the reference 4th quintile (the lowest-prevalence group of cognitive impairment). The adjusted OR of the 2nd quartile of METs scores was significantly lower than the reference quartile. Regarding alcohol consumption, adjusted ORs of 1–149 and 150–299 (g ethanol/wk) groups were significantly lower than the reference group (non-drinkers or rare drinkers).

**Table 3:** Odds ratios (ORs) of cognitive impairment (MMSE score <24) according to levels of predictor variables.

Predictor variables	Prevalence of cognitive impairment (%)	Unadjusted OR (95%CI)	Adjusted OR^a^ (95%CI)
BMI (kg/m^2^)		P = 0.2283	P = 0.6833
1st Q (<20.7)	15/361 (4.2)	0.70 (0.35–1.37)	0.77 (0.38–1.55)
2nd Q (20.7–22.1)	24/360 (6.7)	1.15 (0.63–2.10)	1.08 (0.58–2.02)
3rd Q (22.2–23.5)	21/358 (5.9)	1 (reference)	1 (reference)
4th Q (23.6–25.1)	25/365 (6.9)	1.18 (0.65–2.15)	1.07 (0.59–1.97)
5th Q (25.2-)	23/357 (6.4)	1.11 (0.60–2.04)	0.98 (0.53–1.84)
Long-term weight change^b^ (kg)		P = 0.0603	P = 0.1472
1st Q (<-4)	22/278 (7.9)	3.31 (1.50–7.32)	2.70 (1.18–6.20)
2nd Q (-4, 0)	21/372 (5.7)	2.31 (1.04–5.11)	2.37 (1.04–5.37)
3rd Q (+1, +3)	13/293 (4.4)	1.79 (0.75–4.25)	1.77 (0.74–4.26)
4th Q (+4, +7)	9/356 (2.5)	1 (reference)	1 (reference)
5th Q (≥+8)	21/380 (5.5)	2.26 (1.02–4.99)	2.24 (0.99–5.04)
METs score (/d)		P = 0.0026	P = 0.2390
1st Q (<21.4)	25/452 (5.5)	1 (reference)	1 (reference)
2nd Q (21.4–27.8)	14/454 (3.1)	0.54 (0.28–1.06)	0.42 (0.21–0.85)
3rd Q (27.9–37.7)	30/450 (6.7)	1.22 (0.71–2.11)	0.87 (0.49–1.54)
4th Q (37.8)	42/456 (9.2)	1.73 (1.04–2.90)	1.05 (0.61–1.81)
Smoking (cigarettes/d)		P = 0.2273	P = 0.6655
0 (Non-smoker)	62/1139 (5.4)	1 (reference)	1 (reference)
0 (Past smoker)	33/486 (1.8)	1.27 (0.82–1.96)	0.77 (0.40–1.50)
<20	10/83 (12.1)	2.38 (1.17–4.84)	2.38 (0.98–5.77)
≥20	5/100 (5.0)	0.91 (0.36–2.33)	0.86 (0.28–2.64)
Alcohol consumption (g ethanol/wk)		P = 0.4888	P = 0.4796
Non-drinker or rare drinker	59/799 (7.4)	1 (reference)	1 (reference)
1–149	19/561 (3.4)	0.44 (0.26–0.75)	0.44 (0.24–0.79)
150–299	8/181 (4.4)	0.58 (0.27–1.24)	0.35 (0.14–0.87)
300–449	14/158 (8.9)	1.22 (0.66–2.24)	0.66 (0.30–1.45)
≥450	11/109 (10.1)	1.41 (0.72–2.77)	0.76 (0.32–1.81)
Green tea consumption		P = 0.3706	P = 0.0727
<1 (times/wk)	7/99 (7.1)	1 (reference)	1 (reference)
1–6 (times/wk)	20/370 (5.4)	0.75 (0.31–1.83)	0.74 (0.29–1.88)
1–3 (cups/d)	48/702 (6.8)	0.96 (0.42–2.19)	0.75 (0.32–1.77)
≥4 (cups/d)	28/598 (4.7)	0.65 (0.27–1.52)	0.48 (0.19–1.19)
Coffee consumption		P = 0.3958	P = 0.3853
<1 (times/wk)	21/347 (6.1)	1 (reference)	1 (reference)
1–6 (times/wk)	38/598 (6.4)	1.05 (0.61–1.83)	1.10 (0.63–1.94)
1–3 (cups/d)	34/738 (4.6)	0.75 (0.43–1.31)	1.20 (0.66–2.17)
≥4 (cups/d)	5/71 (4.7)	1.18 (0.43–3.23)	2.13 (0.71–6.40)
Sleep time (hours/day)		P = 0.0004	P = 0.0726
<6	5/86 (5.8)	1 (reference)	1 (reference)
6	21/533 (3.9)	0.66 (0.24–1.81)	0.67 (0.23–1.94)
7	38/685 (5.6)	0.95 (0.36–2.49)	0.84 (0.31–2.27)
8	35/412 (8.5)	1.50 (0.57–3.96)	1.02 (0.37–2.80)
≥9	12/93 (12.9)	2.40 (0.81–7.12)	1.84 (0.54–6.29)

CI, confidence interval; BMI, body mass index; MET, metabolic equivalent.

^a^ Adjusted for age, sex, education levels, and histories of stroke and diabetes.

^b^ Current weight minus weight at 20 years of age.

**Fig 1:**
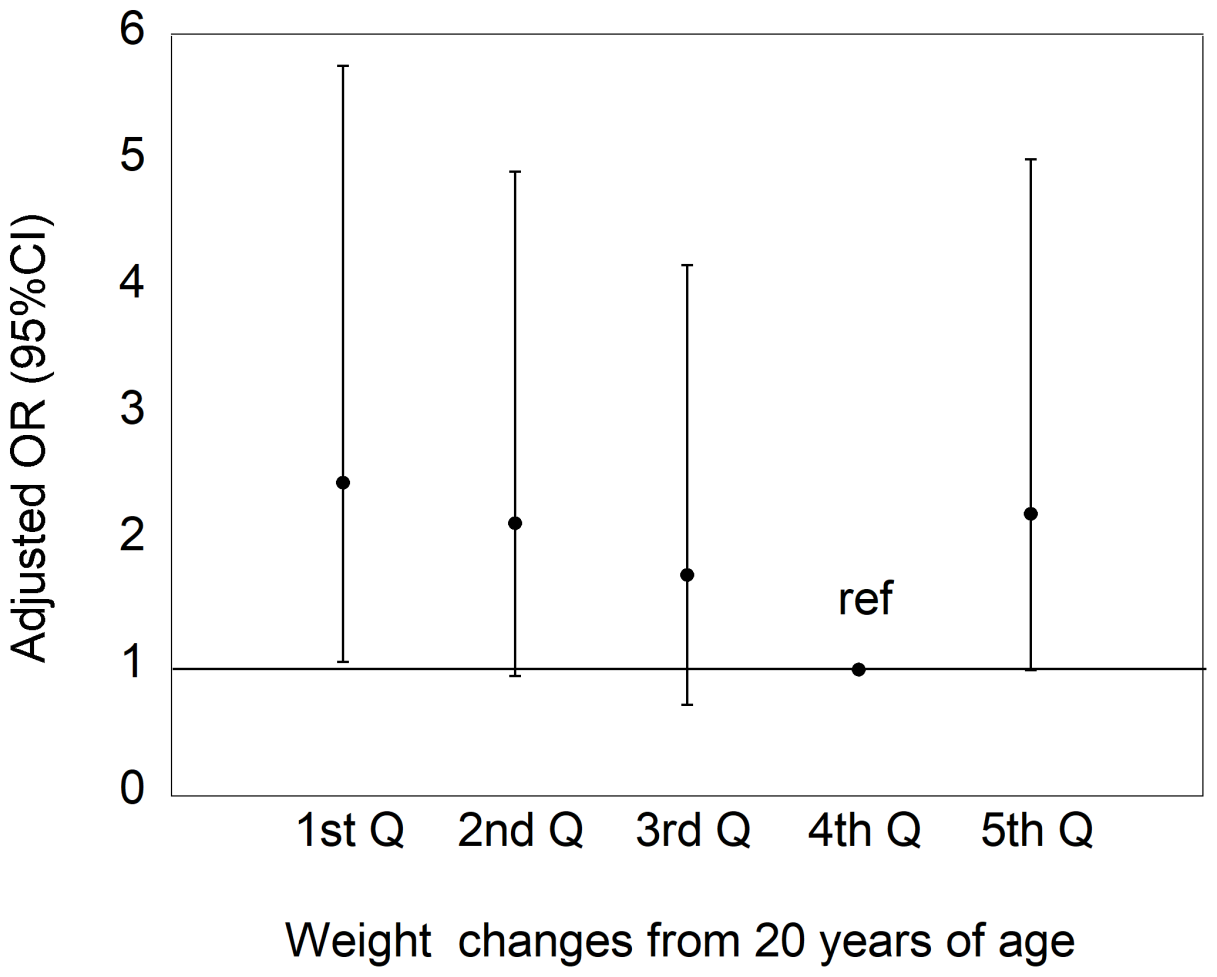
Odds ratios (ORs) of cognitive impairment (MMSE score <24), adjusted for age, sex, education level, and histories of stroke and diabetes, according to quintiles of weight changes from 20 years of age, after excluding 59 individuals who reported rapid weight loss of >4 kg in the past year. Cutoff points of weight changes are 1st Q (<-4), 2nd Q (-4, 0), 3rd Q (+1, +3), 4th Q (+4, +7), and 5th Q (≥+8). P for trend across 1st-4th quintiles was 0.0114.

We further analyzed the association between long-term weight loss and cognitive impairment by excluding 59 individuals who reported rapid weight loss (<-4 kg) in the past year. The association between weight changes from 20 years of age and the prevalence of cognitive impairment is shown in Fig 1, and reflects that shown in Table 3. The adjusted OR of the lowest quintile was significantly higher (OR = 2.48, 95%CI, 1.06–5.77, P = 0.0353) than the reference 4th quintile. In addition, more weight loss quintiles had a higher adjusted OR of cognitive impairment (P for trend = 0.0114) across the 1st-4th quintiles. In contrast, the OR of the highest 5th quintile tended to be higher (OR = 2.24, 95%CI, 0.99–5.04, P = 0.0528) than the reference 4th quintile. When subjects were limited to those aged <75 years, the patterns of association between long-term weight loss and cognitive impairment remained similar.

## Discussion

The present study was the first to thoroughly investigate modifiable factors associated with cognitive impairment and to demonstrate an association between long-term weight loss and cognitive impairment in a Japanese population.

Some studies have shown that low BMI is a risk factor for dementia or cognitive impairment [2,11,21–23]. Recently, a large cohort study showed that underweight individuals (BMI <20 kg/m^2^) are at an increased risk of dementia in all age groups ≥40 years [22]. In a previous study, we reported that low BMI was associated with high cognitive impairment in a population of Japanese outpatients [11].

The present study demonstrated that those with greater weight loss after 20 years of age had higher ORs of cognitive impairment. This suggested that a long-term tendency toward weight loss is a risk factor for cognitive impairment later in life. A few longitudinal studies conducted in the US reported similar findings. For example, Buchman et al. [21] showed that weight loss (BMI decrease) is associated with an increased risk of Alzheimer’s disease during a 5.5-year follow-up of 820 individuals (mean age, 75 years). In addition, Beydoun et al. [2] showed that a 5-year weight loss (BMI decrease, 10th percentile) increases risk of Alzheimer’s disease in middle-aged women (n = 2322). Alhurani et al. [23] conducted a prospective study targeting 1895 men and women aged between 70 and 89 years, and found that greater weight loss from midlife is associated with an increased risk of mild cognitive impairment (MCI, an intermediate stage between normal aging and dementia). Our findings are consistent with those from these three studies, even though the definitions of weight change were not uniform.

While the exact mechanism to explain the effects of weight loss on cognitive function is unclear, some potential mechanisms have been proposed. Vidoni et al. [24] showed that lower BMI is associated with biomarkers of cerebral amyloid and tau in cognitively normal and MCI individuals, suggesting a correlation between lower BMI and higher pathophysiology of Alzheimer’s disease in its earliest stages. Burns et al. [25] reported that less lean body mass is associated with brain atrophy, possibly via systemic anabolic and inflammatory abnormalities associated with Alzheimer’s disease. Furthermore, weight loss may be associated with decreases in body fat mass, a protective factor for cognitive impairment, via a decrease in endogenous estradiol derived from visceral adipocytes [26].

The present study revealed that the quintile with the greatest weight change (the 5th quintile group) also had a tendency toward increased OR (marginal significance) of cognitive impairment, and as a result, the association between weight change and ORs of cognitive impairment was a reverse J-shaped curve. A meta-analysis by Anstey et al. [8] showed a U-shaped association between BMI and later risk of dementia, which was highly consistent with our findings. Several studies have found strong evidence that obesity in midlife has been recognized as a more convincing risk factor for dementia than leanness [9,27]. Obesity is prevalent worldwide, but less prevalent in Japan. In the present study population, the mean BMI was 23.0 kg/m^2^, and percentages of overweight and obese individuals were 21.1% and 1.6%, respectively. Therefore, weight gain may be less important than weight loss as a risk factor for cognitive impairment of the present Japanese population.

We showed that the prevalence of MMSE-assessed (<24) cognitive impairment was 6.2% (mean age, 68.5 years). Some other population-based studies conducted in Japan examined the same outcome measure, and found that the prevalences of cognitive impairment (same definition as the present study) were 7.4% (mean age, 74.5 years) [5], 7.9% (mean age, 72.6 years) [28], and 5.3% (mean age, 73.0 years) [29], which are consistent with our study results.

Individuals who consumed less than 300 g ethanol/week (equivalent to 43 g ethanol/day or 1.6 *gou* of Japanese *sake*/day) had a lower risk of cognitive impairment than those who did not. It has been widely believed that light-to-moderate alcohol consumption may protect against dementia. A very recent meta-analysis has concluded that modest alcohol consumption (≤12.5 g ethanol/day) is associated with a reduced risk of dementia, while excessive drinking (≥38 g ethanol/day) may elevate the risk [30]. Our present study findings were in line with those from this meta-analysis, but should be interpreted with caution. The influence of alcohol drinking on cognitive function may differ by various factors, such as alcohol type, drinking pattern, and genetic predisposition, in addition to the amount consumed [26]. Therefore, with regard to prevention of dementia, alcohol consumption may not be recommended.

Although physical activity is known to be associated with incident dementia [2], the present study did not find such an association. The METs index reflects total amount of general physical activity, which may not predict cognitive impairment; instead, indices of specific physical activity may better predict cognitive impairment, and should be explored in future studies.

This study has some limitations. First, current weight, weight at 20 years of age, and lifestyle information were self-reported, which may have led to misclassification bias. If this were the case, then the strength of association between lifestyle variables, including body weight, and cognitive impairment were underestimated. Second, there may have been causes of weight loss after 20 years of age which could not be assessed in this study. Such factors may potentially have had a greater impact on later-life cognitive status than the actual weight loss itself, and thus should be investigated in future studies. Finally, MMSE-assessed cognitive impairment was used as the outcome measure of this study. Although MMSE is an appropriate screening tool of dementia with good sensitivity and specificity and has been validated in Japanese [31], MMSE-assessed cognitive impairment includes not only clinically diagnosable dementia but also other types of cognitive impairment, such as MCI [32] and temporary cognitive decline. This could also confer misclassification bias. Therefore, similar epidemiologic studies to ours using more sensitive outcomes reflecting dementia or Alzheimer’s disease should be conducted in the future.

In conclusion, long-term weight loss is associated with cognitive impairment in middle-aged and elderly Japanese individuals. Because the present study used retrospective information, studies using a prospective design should be conducted to confirm this association.
